# Energy Balance Indicators during the Transition Period and Early Lactation of Purebred Holstein and Simmental Cows and Their Crosses

**DOI:** 10.3390/ani11020309

**Published:** 2021-01-26

**Authors:** Deise Aline Knob, André Thaler Neto, Helen Schweizer, Anna C. Weigand, Roberto Kappes, Armin M. Scholz

**Affiliations:** 1 Ludwig Maximilians Universität München (LMU), Tierärztliche Fakultät, Lehr- und Versuchsgut Oberschleißheim, 85764 Oberschleißheim, Germany; Helen.Schweizer@lvg.vetmed.uni-muenchen.de (H.S.); Anna.Weigand@lvg.vetmed.uni-muenchen.de (A.C.W.); Armin.Scholz@lvg.vetmed.uni-muenchen.de (A.M.S.); 2 Programa de Pós-Graduação em Ciência Animal, Centro de Ciências Agroveterinárias, Universidade do Estado de Santa Catarina—CAV/UDESC, Lages CEP 88.520-000, Brazil; andre.thaler@udesc.br (A.T.N.); roberto.kappes@edu.udesc.br (R.K.)

**Keywords:** back fat thickness, BHBA, crossbreeding, Holstein × Simmental, NEFA, rotational cross

## Abstract

**Simple Summary:**

Dairy cows undergo a very challenging time between the weeks immediately before calving and the start of lactation after calving. In particular, high yielding dairy cows, such as purebred Holstein cows, have to cope with a severe negative energy balance. In comparison to the feed (energy) intake, they produce a great surplus of milk energy. The energy deficit is supposed to be smaller in dual-purpose breeds, such as (German) Simmental. Therefore, crossbreeding of both breeds, with the aim of using the advantageous characteristics of both breeds, and the expected advantage of crossbred cows, might reduce the negative effects of the metabolic and physiologic challenges by improving the production efficiency of dairy herds. After calving, Simmental cows and cows with greater Simmental proportions decreased less in the body condition score, had lower concentrations of ketone bodies, and nonesterified fatty acids in the blood, which are common indicators of metabolic disorders during the transition period. In particular, first generation (F1) crossbred cows produced more energy corrected milk (ECM) than purebred Holstein cows, while the other crossbred generations still showed positive heterosis effects for ECM. That means, they produced more ECM than the average of both parental breeds.

**Abstract:**

Crossbreeding in dairy cattle has been used to improve functional traits, milk composition, and efficiency of Holstein herds. The objective of the study was to compare indicators of the metabolic energy balance, nonesterified fatty acids (NEFA), beta-hydroxybutyrate (BHBA), glucose, body condition score (BCS) back fat thickness (BFT), as well as milk yield and milk composition of Holstein and Simmental cows, and their crosses from the prepartum period until the 100th day of lactation at the Livestock Center of the Ludwig Maximilians University (Munich, Germany). In total, 164 cows formed five genetic groups according to their theoretic proportion of Holstein and Simmental genes as follows: Holstein (100% Holstein; *n* = 9), R1-Hol (51–99% Holstein; *n* = 30), first generation (F1) crossbreds (50% Holstein, 50% Simmental; *n* = 17), R1-Sim (1–49% Holstein; *n* = 81) and Simmental (100% Simmental; *n* = 27). The study took place between April 2018 and August 2019. BCS, BFT blood parameters, such as BHBA, glucose, and NEFA were recorded weekly. A mixed model analysis with fixed effects breed, week (relative to calving), the interaction of breed and week, parity, calving year, calving season, milking season, and the repeated measure effect of cow was used. BCS increased with the Simmental proportion. All genetic groups lost BCS and BFT after calving. Simmental cows showed lower NEFA values. BHBA and glucose did not differ among genetic groups, but they differed depending on the week relative to calving. Simmental and R1-Sim cows showed a smaller effect than the other genetic groups regarding changes in body weight, BCS, or back fat thickness after a period of a negative energy balance after calving. There was no significant difference for milk yield among genetic groups, although Simmental cows showed a lower milk yield after the third week after calving. Generally, Simmental and R1-Simmental cows seemed to deal better with a negative energy balance after calving than purebred Holstein and the other crossbred lines. Based on a positive heterosis effect of 10.06% for energy corrected milk (ECM), the F1, however, was the most efficient crossbred line.

## 1. Introduction

Crossbreeding in dairy cattle serves mainly for the improvement of functional traits and milk composition of Holstein herds [1,2,3,4,5,6] or uses beef-breed sires over dairy cows to increase income from sales of the beef-cross–dairy calves born on the dairy farm [7]. The majority of these studies, however, report only the results of the first crossbreeding generation (F1) in comparison with the purebred line(s), such as e.g., [8,9] for Holstein-Jersey, or [10] for Holstein-Montbeliarde and Holstein–Viking Red, [11] for Holstein-Gir, or [12] for Holstein-Simmental cows. Only the F1 can reach the maximum heterosis effect, which might improve the performance for most trait complexes, e.g., productivity, efficiency, reproduction, and/or vitality of the F1 offspring by surpassing the average of the parental lines [13]. The performance of the subsequent breeding generations in dairy cattle crossbreeding programs stays often unanswered or is not being communicated anymore. In dairy cattle, if at all [14], three different crossbreeding approaches are being used most frequently. (1) Backcrossing, is more related to pure breeding because after crossing with a foreign breed or line, the subsequent matings occur again only within the original parental breed or line leading to a refined breed (with some foreign blood). German Simmental breeders, for example, use Red Holstein semen to refine the parental breed “Deutsches Fleckvieh” (= German Simmental). (2) Three-breed rotational crossbreeding works with three dairy breeds [15]. After producing the first F1 generation from two breeds, a third breed is crossed in leading again to a theoretical “F1” offspring generation that retains the expected maximum individual (100%) heterosis level also for the second crossbreeding generation, as described by Clasen et al. [16] for a terminal crossbreeding program, with two dairy breeds and one beef breed. While following crossbreeding generations, by applying a sire rotation of three breeds (lines), the heterosis effect will reach a level of 85.7% of the maximum heterosis effect to be expected in the F1 generation (e.g., https://www.crv4all.de/service/procross-genetik/). (3) The simplest approach is a two-breed rotational cross leading to a large variety of the genetic proportions of the two parental breeds in the crossbred population with an average heterosis effect of 66.6% of the expected maximum. The third approach, for example, is a standard procedure in the New Zealand “Kiwi Cross” breeding program by crossing New Zealand Holsteins with New Zealand Jerseys (https://www.lic.co.nz/products-and-services/artificial-breeding/crossbreeding-kiwicross/) [17,18].

A simulation study showed that terminal and rotational crossbreeding strategies using Swedish Red and Swedish Holstein cows can improve profitability in average Swedish organic and conventional dairy herds with purebred Swedish Holstein. The largest economic benefits were shown for rotational crossbreeding, where all animals in the herd were crossbreds and expressed averagely 67% (66.6%) of the full heterosis [16].

Generally, independent of belonging to purebred or crossbred lines, dairy cows usually need to mobilize body fat reserves during early lactation to be able to meet the substantial energy demands for milk synthesis [19]. The evaluation of body condition scores (BCS) is one technique that can be used to visually and subjectively estimate the intensity of the loss of the subcutaneous fat reserves [20]. Ultrasound back fat thickness (BFT) is a (more) objective and direct measure for the evaluation of subcutaneous fat [21]. Both BCS and BFT may be, therefore, used to evaluate the energy status of the cow [22]. Differences for these variables between breeds are reported especially by comparing dual-purpose breeds or crossbreds with Holstein cows [1,15,23].

During the process of using body tissues as an energy source, the metabolic status and blood parameters of the cow change. For example, nonesterified fatty acids (NEFA) and beta-hydroxybutyrate (BHBA) are being generated [22,24]. By comparing Holstein, Brown Swiss, Simmental, and crossbred Holstein × Simmental cows, Blum et al. [25] observed higher NEFA values in Holstein cows. They attribute this observation to the comparably higher milk yield and consequently increased mobilization of body reserves. In addition, they observed higher NEFA concentrations at the beginning of the lactation caused by the high milk yield. In the study by Mendonça et al. [4], no differences were observed between Holstein and crossbred F1 Holstein × Montbeliarde cows for NEFA and BHBA values. Sgorlon et al. [26] reported also no difference for NEFA and BHBA concentrations by comparing Holstein and Simmental cows after the lactation peak.

In addition, according to findings by Knob et al., Nolte, and Diepold [1,2,3,27,28], crossbred F1 Holstein × Simmental cows show a better reproductive performance with similar milk yields in comparison with the parental Holstein breed.

This study was performed to better understand and to compare the crossbred generations following the F1 in a two-breed rotational system with the parental dairy cattle breeds Holstein and Simmental (German Fleckvieh—a dual-purpose breed). The majority of studies evaluating the transition period of dairy cows were performed with the Holstein breed [29,30,31]. A number of studies evaluated Holstein × Montbeliarde, Holstein × Jersey or Holstein × Simmental crossbred cows in comparison with one of the parental breeds (most often Holstein) [4,32,33,34]. Only a very few studies compared crossbred Holstein × Simmental cows and both parental breeds [12,35]. Scata et al. [12], for example, compared the immunologic status of the cows. They included, however, only the F1 generation and both parental breeds. Our study aimed especially at the evaluation of the prepartum period and the first 100 days of lactation, the time when the cows are more susceptible to a challenging negative energy balance and the related negative effects. By comparing different genetic groups, especially by including the crossbred generations after the F1, the study can provide new insights not only about the energy balance indicators, but also about the performance of the genetic groups in comparison to the parental breeds. This study hypothesized that the higher the Simmental proportions the better cows can pass the transition period combined with a smaller negative effect on BCS and BFT (loss) and more favorable blood parameters indicating only a minor negative energy balance. Therefore, the objective of the study was to compare indicators of the metabolic energy balance (BHBA, NEFA, glucose, BCS, BFT) as well as of milk yield and milk composition of Holstein and Simmental cows and their crossbred stall mates from the prepartum period until the 100th day of lactation.

## 2. Materials and Methods

All procedures carried out with animals in the present study were approved by the animal ethical committee of the Government of Upper Bavaria under the protocol number ROB-55.2-2532.Vet_03-18-60.

### 2.1. Animals and Management

The study was carried out at the Livestock Center of the Ludwig Maximilians University (Munich, Germany). The herd consisted of about 120 lactating cows of the Holstein and Simmental breeds as well as their crosses. The crossbred cows were the result of a rotational crossbreeding system that started in the year 1999. They carried different genetic proportions of the Simmental and Holstein breed. Therefore, cows were divided into 5 genetic groups according to their theoretic proportion of Holstein and Simmental genes, as follows: Holstein (100% Holstein; *n* = 9 cows), R1-Hol—between (51 and 99% Holstein; *n* = 30 cows), F1 crossbreds (50% Holstein and 50% Simmental; *n* = 17 cows), R1-Sim (between 1 and 49% Holstein; *n* = 81 cows) and Simmental 100% Simmental; *n* = 27 cows). Twenty-two cows entered the study two times in two subsequent lactations (R1-Hol: *n* = 3, F1: *n* = 3, R1-Sim: *n* = 10, Simmental: *n* = 6). For the study, all lactating cows available in the herd independent of their genetic group were used. The small number of purebred Holstein (*n* = 9) included all available purebred Holstein cows during the study time. A modified classification of the genotypes was not considered in order to achieve a clear definition of purebred, F1, and R1 cows.

The lactating animals were kept in a confinement system throughout the entire year. The free-stall barn was divided into two parts. One side of the free stall was equipped with a straw deep box bedding system and the other side used soft rubber lying mats as a bedding system. The cows were randomly housed at one of both sides so that the proportion of each genetic group and parity was similar on both sides. In each part of the barn, cows had free access to an automatic milking system (AMS) from Lely Industries N.V. Maasland/Netherlands (Astronaut A3 and Astronaut A3 next). Cows entered the AMS through an electronic identification system positioned in the neck collars. Information regarding milking frequency, milk yield, milk composition indications, somatic cell count (SCC), and body weight (BW) were recorded daily and per milking. SCC and milk composition were estimated quarter wise by the optional “Milk Quality Control System” MQC-C. The SCC test is based on the drain time of a mixture of the milk and “Astri-Cell” (a fluid needed for measuring the somatic cell count in the milk) and measures the viscosity of the milk sample. Fat and protein percentages are so called indications, because the light used by the MQC-C to calculate fat and protein “indications” corresponds to a certain lipid droplet (fat) globule size. The milk solid indicators (fat and protein in %) provided by the MQC-C were based on the calibration by the monthly reference (performance test) data of the Landeskuratorium der Erzeugerringe für tierische Veredelung in Bayern e.V. (LKV). Generally, the daily data provided by the AMS system should have been more accurate than the monthly performance test data originating from the LKV, though they might have been slightly biased. This bias (in comparison to the LKV data), however, was equal for all cows. Data regarding milk composition indications and SCC estimates provided by the MQC-C were previously validated for research purposes [36].

The diet offered to the lactating cows was a partial mixed ration composed of corn silage (38.47% in dry matter = DM), grass and clover silage (33.39% in DM), hay or barley straw (4.85% in DM), concentrates (corn whole meal = 9.92% in DM, wheat whole meal = 2.48% in DM, barley whole meal = 2.48% in DM, rape seed whole meal = 6.34% in DM, molasses = 1.32% in DM), livestock salt = 0.11% in DM, and a mineral mix (SALVANA 1104 prenatal beta, Wertingen, Germany; 0.64% in DM) with an average net energy of lactation of 6.45 MJ per kg DM and a usable raw protein content of 142 g/kg DM in order to meet the nutrient and energy requirements for a daily milk yield of 24 kg with 4.0% fat and 3.4% protein. Lactating cows received additional concentrates (up to 8 kg as pellets and molasses) at the AMS according to their average milk yield during the last milking and days in milk (DIM). Dry cows received a modified ration consisting of corn silage (26.47% in DM), grass and clover silage (53.54% in DM), barley straw (18.83% in DM), a mineral mix (SALVANA 1104 prenatal beta, Wertingen, Germany; 1.079% in DM), and livestock salt (0.08% in DM) with an average net energy of lactation of 5.37 MJ per kg DM and a usable raw protein content of 118 g/kg DM. These are only example rations, because the rations were adapted as soon as new corn or grass silage batches were opened. Corn and grass silage were stored in drive-in (slap) silos or in bale silos. All rations were based on chemical analysis results of all components.

The yearly milk performance of the herd was 11,114 kg for purebred Holstein cows (with an average age of 5.1 years, and 2.7 lactations) and 9122 kg for purebred Simmental cows (with an average age of 3.7 years, and 1.6 lactations). The crossbred cows reached an average milk yield of 10,728 kg per lactation (with an average age of 4.4 years, and 2.1 lactations). The age at first calving differed only slightly between 25.7 and 26.3 months among the five groups, while the time between calvings varied between 378 days for purebred Simmental, 436 days for purebred Holstein, and intermediate values for crossbred cows (~389 days). A slightly different variation showed the number of services per pregnancy with 2.13 for purebred Simmental, 2.25 for purebred Holstein, and 1.81 for crossbred cows. Clinical mastitis incidence varied between 28% for purebred Simmental, 31.99% for Holstein, and 30.19% for crossbred cows, respectively.

The cows underwent a dry off period of 60 days before the expected calving date. Before dry off, milk samples of cows with SCC higher than 200,000 cells/mL was collected and incubated on sheep blood agar in order to perform an antimicrobial susceptibility testing (Benestermycin, Mast Group, Ltd., Merseyside, U.K., and Oxoid™ Cloxacillin, Fisher Scientific, Arendalsvägen, Göteborg—Sweden). The antibiotic treatment was performed based on the test result. Cows with SCC lower than 200,000 cells/mL and no udder health problems during lactation received no antibiotic treatment. They were treated only with an internal teat sealer in order to prevent the development of new intramammary infections (Ubroseal^®^ Dry Cow, Boehringer Ingelheim Animal Health UK Ltd., Berkshire, UK).

### 2.2. Data Collection

The study was carried out between April 2018 and August 2019. All multiparous and primiparous cows of the farm were evaluated 21 days before the expected day of calving until day 100 of lactation (the first 14 weeks after calving). Data recording and taking samples of each cow participating in the study took place in a weekly routine. Body condition score (BCS), back fat thickness (BFT), locomotion score (LS), blood parameters, such as beta-hydroxybutyrate (BHBA), glucose, and non-esterified fatty acids (NEFA), served as study traits. A visual score from 1 (very thin cow) to 5 (very fat cow) according to Ferguson et al. [37] served as the basis for the BCS. On the day of body condition scoring, BFT was measured with an ultrasonic device (KX5200, Kaixin Electronic Instrument CO, Xuzhou, Jiangsu, China) using a linear probe (6.5 MHz). The measurement point lay on an imaginary line between the tuber ischia and the tuber coxae about 10 cm cranial of the tuber ischia [21]. After freezing the suitable B mode ultrasound image, the layer of subcutaneous fat was measured to the nearest millimeter. To minimize potential errors, only one person performed all measurements. The BFT was always assessed on the right body side of the cow. Similarly, with a weekly routine, blood was sampled from the coccygeal vein of each cow using serum tubes equipped with a vacuum system and a clot activator in combination with curaVet^®^ Easy-Lance needles for single use (1.2 × 40 mm) from Wirtschaftsgenossenschaft deutscher Tierärzte eG (WDT, Garbsen, Germany). After 3 h, the blood samples were centrifuged for the serum separation at 3000 rpm for 10 min. BHBA and glucose concentrations were additionally tested at the time of blood sampling. A portable measurement device from Pharmadoc (Lüdersdorf, Germany) served as a tool for the determination of the BHBA concentration (mmol/L). The glucose (mg/dL) concentration was measured using the portable ACCU-CHEK Guide device (Roche Diabetes Care Deutschland GmbH). The serum samples provided the basis for the NEFA (mmol/L) evaluations using commercial test kits from Diaglobal^®^ (Berlin, Germany). After the analysis, the serum samples were frozen at −20 °C.

Locomotion scoring (LS) was carried out with the help of a 5-point scoring system [38] by looking at the cow while standing and walking. Cows received scores between 1 (no lameness, back straight in standing and walking, normal treading) and 5 (severe lameness, back is bent in walking and standing, with one or more legs only partially or no treading). The LS was always performed by the same researcher, previously trained to perform the evaluations.

### 2.3. Statistical Analysis

The information of eight cows (Simmental = 4, R1-Sim = 3, Holstein = 1) was excluded from the final data set because of having less than 3 data records after calving (not adapted to the AMS, died, or were culled before this period). Data were classified according to the week relative to the calving date. Parity was grouped as first, second, and third or more parturitions. To obtain the normality of data, SCC was transformed into somatic cell score (SCS) by the logarithmic scale applying the following equation [39]:

SCS = log2 (SCC/100,000) + 3(1)

The equation:

ECM = (0.327 × MY) + (12.95% × F × MY/100) + (7.65% P × MY/100)(2)

where MY = milk yield in L/day, F = fat percentage, and *p* = protein percentage] provided the energy corrected milk yield (ECM in kg) [40]. A restricted maximum likelihood (REML) analysis of variance using the mixed model procedure of SAS 9.4.was performed. The REML analysis is especially suited for unbalanced data as present in the study [41]. The mixed model contained the fixed effects: genetic group (i = 1–5), week relative to calving (j = 1–17; −3 to 14), the interaction between genetic group and week relative to calving (i × j), parity (k = 1–3), calving year (l = 2018, 2019), calving season (m = 1–6), milking (data collection) season (*n* = 1–6, with seasons 1 = January/February, 2 = March/April…, 6 = November/December), bedding system (o = 1, 2), and the random effect: cow. Data were tested for normality of the residuals using the Kolmogorov–Smirnov test. The significance level was set to *p* ≤ 0.05 both for the results of the variance analysis (F test) as shown in the appendix tables and for the results of the t test comparing the least squares means of the fixed effects including the interaction effects of the model.

Heterosis (H, in %) was calculated according to the formula:

H = (average performance of the crossbred generation/(P1 + P2/2) × 100) −100, with P1 and P2 = average performance of purebred parental generations 1 and 2(3)

or for the F1 crossbred generation:

H = F1/(MPV) × 100 − 100, with MPV (mid parent value) = (P1 + P2)/2(4)

## 3. Results

**Figure 1:**
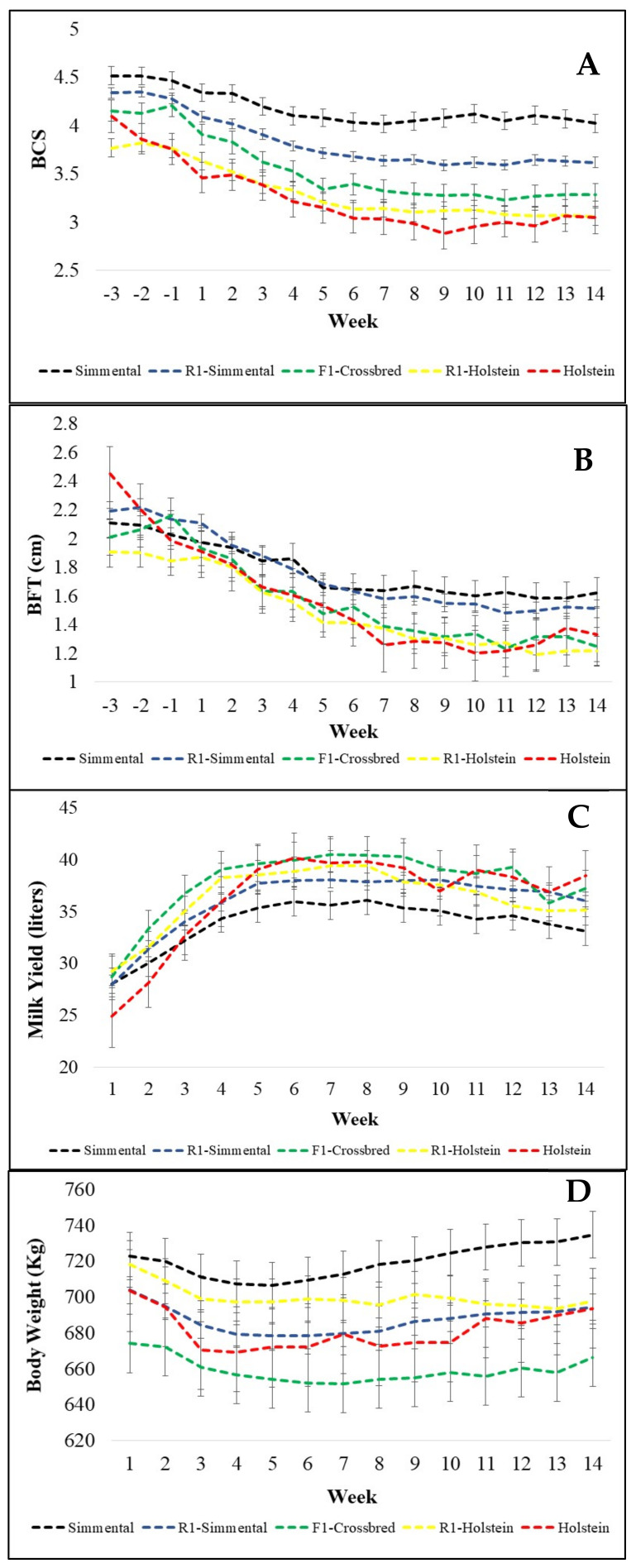
Weekly means of body condition score (BCS) (**A**), back fat thickness (BFT) (**B**), milk yield (**C**), and body weight (**D**) between week three before calving and week fourteen after calving for purebred Holstein, Simmental cows, and their crosses.

The results of the study showed a difference among genetic groups for most of the variables related to the energy balance before calving, until day 100 after calving. For BCS, for example, Simmental and R1-Sim cows have a greater BCS than the other genotypic groups, while cows with <50% Simmental proportions did not differ (Table 1, F test in Appendix A: Table A1). The average difference between the purebred Simmental and Holstein cows reached almost 1 point (Table 1). The genetic groups R1-Hol and Holstein did not differ and had the lowest BCS. The genetic groups differed slightly in BFT with Simmental and R1-Sim showing higher values (*p* = 0.1024, Appendix A: Table A2). All genetic groups declined in BCS and BFT after calving (Figure 1A,B). The lowest BCS occurred around week 6 to 8, while BFT reached the lowest values around week 10. Holstein cows lost more than one BCS point until week 9 after calving representing a 30% drop of the initial BCS. With an increasing Simmental proportion, the BCS loss decreased by 20%, 24%, 18%, and 12% for R1-Hol, F1- crossbred, R1-Sim, and Simmental cows, respectively. The proportional decline in BFT in comparison to the values before calving was higher than the proportional BCS decline for all genetic groups. Holstein cows, for example, lost approximately 45% of (subcutaneous) body fat, while the three groups of crossbred cows lost approximately 35% and Simmental 25% of back fat, respectively (Figure 1B). Holstein, R1-Hol, and R1-Sim cows had an almost similar BW, while F1 crossbreds showed the lowest and Simmental the highest BW (*p* = 0.0482, Appendix A: Table A6; and Table 1). The absolute difference reached approximately 60 kg BW (Figure 1D).

The genetic groups yielded similar amounts of milk (Table 2). There was, however, an interaction between genetic group and week (*p* < 0.0001, Appendix A: Table A8, and Figure 1C). After the fourth week of lactation, Simmental yielded a lower amount of milk than did the other genetic groups. The five genetic groups reached the lactation peak between weeks 6 and 8 (Figure 1C) with Simmental yielding the lowest amount of milk or ECM in comparison to the other genetic groups (Table 2). All genetic groups showed a similar milk fat percentage (*p* = 0.1328, Appendix A: Table A10). Based on the lowest milk yield, Simmental cows, however, produced the smallest amount of milk fat (Table 2). In contrast to the milk fat percentage, Simmental cows showed the greatest milk protein percentage (Table 2) leading to equal amounts of protein for the genetic groups (*p* = 0.4783, Appendix A: Table A12). In addition, it became obvious that F1 cows performed—in tendency—best for the traits: milk yield, ECM, milk fat in kg and %, milk protein in kg and % (Table 2). Unexpectedly, however, F1 cows had only an intermediate SCS, while cows with a Simmental proportion of > 50% showed the lowest SCS (Table 2).

**Figure 2:**
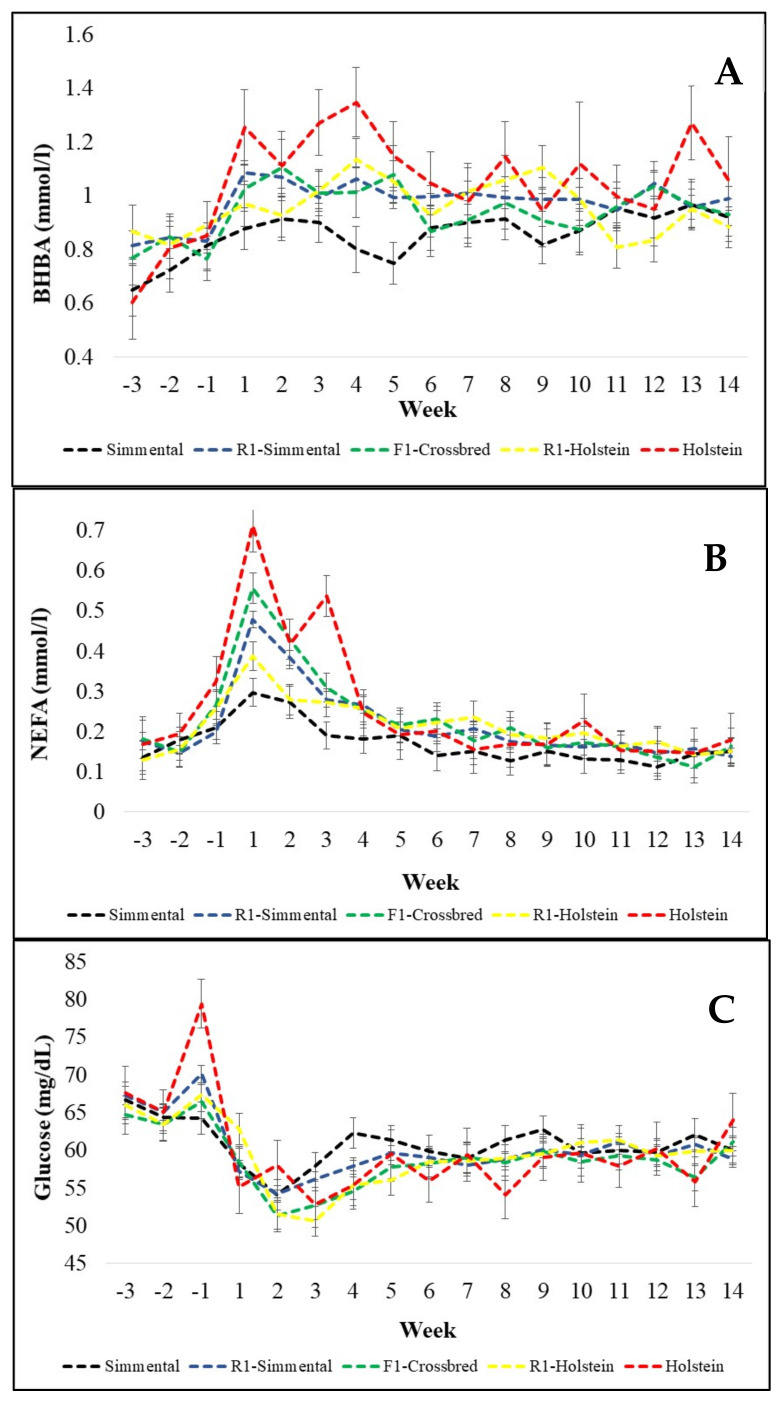
Weekly means of beta-hydroxybutyrate (BHBA) (**A**), nonesterified fatty acids (NEFA) (**B**), and glucose (**C**) between week three before calving and week fourteen after calving for purebred Holstein, Simmental cows, and their crosses.

The blood parameters evaluated as indicators of the energy balance differed significantly among genetic groups—except for glucose (Appendix A: Table A3, Table A4 and Table A5). The week relative to calving affected all traits observed—except the amount of milk fat (Appendix A: Table A1, Table A2, Table A3, Table A4, Table A5, Table A6, Table A7, Table A8, Table A9, Table A10, Table A11, Table A12, Table A13 and Table A14). Simmental cows had significantly lower NEFA values than all other genetic groups (Table 1). In addition, NEFA concentrations increased already slightly in the week before calving and reached peak values during the first week after calving (Figure 2B) combined with a significant interaction between genetic group and week (*p* = 0.0029, Appendix A: Table A4). During the first week after calving, Holstein and F1 crossbred cows had higher NEFA values than the other genetic groups. Holstein cows still had higher values during the third week after calving. From week 4 until the end of the experimental period, differences among genetic groups vanished (Figure 2B). BHBA differed significantly among genetic groups (*p* = 0.0229, Appendix A: Table A3), but showed no interaction between genetic group and week (*p* = 0.4630, Appendix A: Table A3) with Simmental showing the lowest level for BHBA (Table 1). Generally, BHBA concentrations increased significantly from the prepartum period until weeks one to four after calving, while Simmental showed the smallest dynamics for BHBA (Figure 2A). Unexpectedly, glucose levels did not differ significantly among breeds and showed no significant interaction between breed and week (*p* = 0.3904 and *p* = 0.3297, respectively; Appendix A: Table A5). Glucose values, however, increased in the week just before calving, especially for Holstein cows, and decreased rapidly during the first and second week after calving (*p* < 0.0001, Appendix A: Table A5). Afterward, glucose concentrations increased slightly until week 4 to 5 after calving and remained stable until week 14 (Figure 2C).

## 4. Discussion

This study analyzed the potential differences among Holstein, Simmental, and their crossbred stall mates regarding the dynamics of the energy balance for the period between week three before calving and week fourteen after calving (100th DIM). The main limitation of this study is the fact that the cows were not equally distributed among genetic groups. Based on the available herd structure, only nine purebred Holstein cows (*n* = 2 first parity) entered the study. In our opinion, however, this uneven data structure did not strongly affect the outcome of the present study. The results of the REML variance analysis (Appendix A) and corresponding t tests for the least squares means of the performance parameters and indicators of energy balance (Table 1 and Table 2) provided reasonable estimates for all genetic groups and the other fixed effects included into the model. The lactation performance of the nine Holstein cows in our study with 11,114 kg milk (305-day performance) surpassed the average milk yield of the purebred Bavarian Holstein cows by 1815 kg milk, while purebred Simmental cows of the Livestock Center reached a lactation milk yield of 9122 kg in comparison to 7955 kg of the purebred Bavarian Simmental cows [42]. Milk solid contents differed also slightly between study cows and the Bavarian populations of purebred Holstein and Simmental cows with fat (kg) = 401.8 vs. 379 or 367.6 vs. 334, and protein (kg) = 370.4 vs. 318 or 318.9 vs. 281, respectively. Therefore, it can be assumed that the purebred cows in our study were representative for the above average cows of Bavarian Holstein and Simmental cows.

By comparing the BCS of purebred Holstein and Simmental cows with crossbred cows, it becomes obvious that the BCS increases in tendency with the Simmental proportion, because Simmental, as a dual-purpose breed, has more body (fat and muscle) reserves than Holstein [26,43]. Therefore, based on the common complementarity effect of crossbreeding (defined as additive genetic effect), crossbred cows have BCS values between both purebred cattle breeds, Holstein, and Simmental. Knob et al. and Ledinek et al. [1,35] reported likewise higher BCS values for crossbred Holstein × Simmental cows in comparison with purebred Holstein cows. Similar to the own results, Ledinek et al. [35] described an increasing BCS with an increasing Simmental proportion by comparing Holstein, Simmental, and crossbred cows with different proportions of Holstein and Simmental genes. The favorable BCS and BFT values, and, especially the lower BCS loss of cows with higher Simmental proportions are possibly related to the different nutrient partitioning between milk production and body reserves [35,44]. Holstein cows allocate more energy to milk production [45]. The lowest BCS and BFT of Holstein cows may reflect the genetic selection for milk yield and dairy type (“milk nobility”), whereas, for dual-purpose breeds, like Simmental, the selection has been focused on body conformation (including potential carcass quality), milk fat, and milk protein production [4].

Cows in our study, however, especially Holstein had a higher BCS than in other studies [1,46] during the prepartum period. Whereas, genetic groups with higher Holstein proportions demand a higher energy supply to be able to meet the energy requirements for milk production. As a consequence, they mobilize more body fat for the conversion into energy leading to higher values of NEFA and BHBA. Šamanc et al. [47] reported that cows that lose more than 0.75 BCS points have higher NEFA values. In agreement with the previous finding, the genetic groups Holstein, R1-Sim, R1-Hol, and F1, which lost more than 0.7 BCS points during the study period (Figure 1A), showed significantly higher NEFA levels than purebred Simmental (Table 1).

Caused by the high milk yield, especially during the first weeks after calving, BCS and BFT decreased in all genetic groups (Figure 1A,B). This pattern is related to the negative energy balance. Immediately after calving, the dry matter intake increases but not as fast as the milk yield, and therefore, to support the energy requirement for milk production, the cows start to use body reserves as energy source [22,48,49]. The body tissue mobilization, especially that of adipose tissue, is converted into energy sources to be used by the cows through hepatic gluconeogenesis [50]. Indicators of this lipomobilization are increasing serum concentrations of NEFA and BHBA (Figure 2A,B) shortly before and immediately after calving [51].

In a review regarding NEFA in dairy cattle, Adewuyi et al. [52] reported that healthy cows usually have NEFA levels below 0.2 mmol/L, while NEFA values increase slowly during the week before calving, finally reaching values between 0.5 and 2 mmol/L during the first week after calving. Afterward, NEFA levels decrease slowly to “normal”, though, values greater than 0.7 mmol/L indicate a severe negative energy balance. Six weeks after calving, NEFA values should again reach levels below 0.3 mmol/L. This pattern of NEFA dynamics occurred also in all genetic groups of our study (Figure 2B). Holstein and F1 crossbred cows, however, showed higher NEFA peaks during the first week after calving than the other genetic groups (Figure 2B), what is possibly related to the higher decline in BCS for these genetic groups (Figure 1A). The period just after calving, is that of the highest energy challenge, due to a relatively low DMI combined with the beginning of lactation [4,49]. The energy requirement of the mammary gland in dairy cows is responsible for 50% to 85% of the whole-body glucose consumption and expands the glucose demand 2.5-fold in the third week of lactation in comparison with the demand during the end of the dry period [53]. Therefore, in our study, the decline of the glucose levels followed the expected pattern during the first three weeks after calving (Figure 2C). The above mentioned increased NEFA values for Holstein and F1 crossbred cows were combined with longer lasting BHBA peak(s) during the following weeks, especially for Holstein cows. A similar pattern was observed by Mendonça et al. [4] comparing Holstein and crossbred Holstein × Montbeliarde cows. Higher NEFA and BHBA concentrations have a negative effect on the immunological status of the cows, especially during the transition period, which is the most challenging and critical time for the health of dairy cows. The immune system of Simmental cows, however, has potentially a more acute response in early lactation, as revealed by the greater expression level of genes involved in the immune system adaptation [54].

Another reason for higher NEFA values with increasing Holstein proportions is that the source of body tissue mobilization of these genetic groups is not always only subcutaneous fat. Cows can also mobilize visceral (abdominal) fat [55]. Therefore, declining BCS and BFT do not completely reflect the body tissue mobilization of the cows. Even with a higher BCS and BFT loss, Holstein and F1 crossbred cows (Table 1, Figure 1A, 1B), might still mobilize other fat depots to fully meet the energy requirements. Weber et al. [56] reported that visceral fat was more readily mobilized in cows in the high yielding group than in the medium and low yielding groups, and thus, visceral fat contributed relatively stronger to elevated NEFA concentrations for high yielding cows. Akter et al. [57] found also that visceral fat depots were more readily mobilized than subcutaneous fat depots in primiparous cows during the first 15 weeks of lactation. The dual-purpose breeds, e.g., Simmental, convert more energy into muscle tissue or fat between muscle fibers, the intermuscular fat depot, instead of storing surplus energy mainly as subcutaneous and visceral fat [58]. Because BCS and BFT measurements aim mainly at the evaluation of subcutaneous fat depots, these traits may not perfectly characterize the energy reserves of a dual-purpose Simmental or crossbred cow.

Glucose levels did not differ among genetic groups. This observation agrees with other studies which compare the glucose levels of Holstein and crossbred cows. Pellizza et al. [33] reported no difference in glucose levels by comparing Holstein and crossbred Holstein × Jersey cows. Glucose records ranged between 59 and 63 (mg/dl) for both genetic groups. Additionally, Blum et al. [25] comparing Holstein, Swiss Brown, Simmental and crossbred Holstein × Simmental cows, Mendonça et al. [4] comparing Holstein and crossbred Holstein × Montbeliarde, and Sgorlon et al. [26] comparing Holstein and Simmental cows, reported no difference in serum glucose concentrations among the genetic groups evaluated. The variation is mainly related to the lactation stage, such as the time prepartum or postpartum, and DIM [49]. Likewise, glucose levels varied depending on the week relative to calving in our study (Figure 2C). At the beginning of lactation combined with a high milk yield, glucose levels are lower due to its uptake to the mammary gland for lactose synthesis. González et al. [59] observed values of 3.37 ± 0.74 (mmol/L) at the beginning of the lactation, and 3.82 ± 0.41 (mmol/L) at the middle third of lactation. Djoković et al. [49] observed values of 2.29 ± 0.48 mmol/L at the beginning of lactation, while at mid-lactation the values ranged from 2.5 to 4.2 (mmol/L). In another study by Djokovic et al. [60], mean glucose levels in cows ranged from 2.2 to 4.0 mmol/L. Cows in the puerperal period, however, had significantly lower blood glucose levels than pregnant cows.

Contrary to other studies, e.g., Schichtl, Brähmig, Ledinek et al., and Nolte [27,35,61,62], our study showed no significant difference for milk yield among the genetic groups—only lower yields for Simmental cows after the third week of lactation. Other research groups reported higher milk yields for Holstein and F1 crossbred cows in comparison to Simmental cows [27,35,61,62]. The genetic selection towards milk yield has been more intensified for the Holstein breed reflecting the better performance for cows with higher Holstein proportions in comparison to Simmental cows [26]. A lower milk yield of Simmental cows in comparison to Holstein cows has been reported in various studies [27,63,64]. Generally, Simmental cows reach approximately 70 to 80% of the milk yield of Holstein cows. An important indicator of the productive efficiency is the ECM, especially because of the adjustment of the milk yield to the proportion of milk solids. Therefore, ECM represents the energy output of cows more accurately than the plain milk yield. The results of our study demonstrated that crossbred cows can be as productive and competitive as Holstein cows without compromising the efficiency of the dairy farm. In agreement with Kargo et al. [65] related to heterosis effects for milk production traits, especially the F1 crossbred generation revealed an unexpected high heterosis effect of 10.06% for the amount of ECM by surpassing the average ECM of the purebred Holstein and Simmental by 3.69 kg/d (Table 2). Even the R1 generations (R1 Holstein, R1 Simmental) reached corresponding heterosis effects for ECM of 5.99% or 1.91%, respectively.

In contrast to other studies, where Simmental cows or crossbred cows have a higher milk fat content than the Holstein cows [3,35,64], there was no difference among the genetic groups in our study. Besides the genetic composition, other factors can affect the milk fat content, e.g., the diet offered to the cows, or the lactation stage. In our study, the similar fat content for all genetic groups may have resulted from the identical diet management for all groups. All cows received the same total mixed ration (ad libitum), while the amount of concentrate provided to the cows in the AMS differed depending on the daily milk yield of the cows. It is discussed, controversially, whether differences in the forage-to-concentrate intake ratio could affect the milk fat content due to changes in the ruminal pH [66]. Another reason, why cows with 50% or more Holstein genes have the same milk fat content like the Simmental cows can be related to the body tissue mobilization. These genetic groups are those having a high initial BCS and showing also a higher BCS and BFT loss. They reach higher NEFA values. This alternative energy source, besides changing the fatty acids profile in milk, can also induce a higher milk fat content. Pires et al. [67] compared the metabolic status with the milk yield and milk composition according to different initial BCS. They reported that cows with higher initial BCS have higher NEFA and BHBA levels. The same group of cows has a higher milk fat content reflecting the availability of body fat.

A large number of studies covering crossbred cows compare the performance of crossbred cows to one of the parental breeds [34,68,69,70], or include only the performance of the F1 generation in comparison to both parental breeds [12,71]. To our knowledge, our study belongs to the very few studies including not only the F1 crossbred generation and both parental breeds, but covers also the crossbred generations following the F1 in a crisscross breeding system like is common for the “Kiwi-Cross” breeding system in New Zealand. The crisscross breeding system in New Zealand, however, includes mainly Holstein and Jersey instead of Holstein and Simmental as presented here. In our opinion, this is the first publication that covers the combination of metabolic traits and some body condition traits with early performance characteristics during the transition period in a crisscross breeding program of Holstein and Simmental.

## 5. Conclusions

Simmental and R1-Simmental cows dealt better with a negative energy balance after calving. These genetic groups lost less back fat and body weight than did the other genetic groups with greater Holstein proportions. In comparison to purebred Simmental, crossbred cows, besides Holstein, yielded greater amounts of milk after the third week of lactation, what caused higher NEFA and BHBA values indicating a stronger body tissue mobilization to meet the energy requirements of lactation. The F1 crossbred cows showed a significant heterosis effect on the amount of ECM of 10%. The use of Simmental semen in a Holstein herd might therefore be an option to improve the metabolic elasticity (capability of adapting to metabolic imbalances) of the cows related to the period just before calving until the 100th lactation day. Even both crossbred generations after the F1 (R1) did not differ significantly in ECM from purebred Holstein, but showed lower peak values for NEFA and BHBA immediately after calving.

## Figures and Tables

**Table 1:** Least squares means (LSM), standard errors of estimation (SEE) and observation numbers (N) for body condition score (BCS), back fat thickness (BFT), beta-hydroxybutyrate (BHBA), non-esterified fatty acids (NEFA), glucose, body weight, and locomotion score (LS) of Holstein, R1-Holstein, first generation (F1)-Crossbred, R1-Simmental, and Simmental cows.

		Holstein *		R1-Holstein		F1		R1-Simmental		Simmental	
Trait	N	LSM	SEE	LSM	SEE	LSM	SEE	LSM	SEE	LSM	SEE
BCS	2724	3.26 ^c^^,d §^	0.14	3.31 ^d^	0.08	3.55 ^c^	0.09	3.83 ^b^	0.05	4.18 ^a^	0.08
BFT (cm)	2721	1.58 ^a,b^	0.16	1.49 ^b^	0.09	1.58 ^a,b^	0.10	1.76 ^a^	0.05	1.77 ^a^	0.09
BHBA (mmol/L)	2005	1.05 ^a^	0.06	0.95 ^a,b^	0.04	0.94 ^a,b^	0.04	0.97 ^a^	0.02	0.85 ^b^	0.03
NEFA (mmol/L)	2004	0.25 ^a^	0.02	0.21 ^a^	0.01	0.22 ^a^	0.01	0.21 ^a^	0.007	0.17 ^b^	0.01
Glucose (mg/dL)	2126	59.9	1.21	59.4	0.79	58.7	0.85	60.2	0.41	60.8	0.71
Body Weight (kg)	15,252	681 ^a,b,c^	21.9	700 ^a,b^	12.6	659 ^c^	16	687 ^b,c^	7.3 ^b^	719 ^a^	12.8
LS	2701	1.67 ^a^	0.18	1.46 ^a,b^	0.10	1.38 ^a,b^	0.12	1.43 ^a,b^	0.06	1.25 ^b^	0.10

^§^ Different superscripts within lines describe significant differences with *p* < = 0.05. * Genetic groups according to their theoretic proportion of Holstein and Simmental genes: Holstein = 100% Holstein, R1-Holstein—between >50 and <100% Holstein, F1-Crossbreds (50% Holstein/50% Simmental), R1-Simmental— >50 and <100% Simmental, and Simmental = 100% Simmental.

**Table 2:** Least squares means (LSM), standard errors of estimation (SEE), and observation numbers (N) for variables of the automatic milking system (AMS) related to milk yield and composition, and somatic cell score (SCS) of Holstein, R1-Holstein, F1-Crossbred, R1-Simmental, and Simmental cows.

		Holstein *		R1-Holstein		F1		R1-Simmental		Simmental	
Trait	N	LSM	SEE	LSM	SEE	LSM	SEE	LSM	SEE	LSM	SEE
Milk yield (L)	15,259	36.4	1.31	36.3	1.34	37.8	1.6	36	0.7	33.8	1.3
ECM (kg) **	14,988	37.9 ^a^^,b §^	2.27	38.9 ^a,b^	1.35	40.39 ^a^	1.65	37.4 ^a,b^	0.75	35.5 ^b^	1.31
Fat (%)	14,992	3.58	0.11	3.71	0.07	3.72	0.08	3.55	0.03	3.52	0.07
Fat (kg/day)	14,988	1.3 ^a,b^	0.08	1.36 ^a^	0.05	1.39 ^a^	0.06	1.26 ^a,b^	0.02	1.19 ^b^	0.04
Protein (%)	14,992	3.26 ^b^	0.06	3.34 ^b^	0.03	3.39 ^a,b^	0.04	3.34 ^b^	0.02	3.48 ^a^	0.04
Protein (kg/day)	14,988	1.19	0.06	1.22	0.04	1.29	0.05	1.20	0.02	1.18	0.04
SCS	15,144	2.88 ^a,b^	0.42	2.93 ^a^	0.25	2.68 ^a,b^	0.30	2.27 ^b^	0.14	2.18 ^b^	0.24

^§^ Different superscripts within lines describe significant differences with *p* < = 0.05. * Genetic groups according to their theoretic proportion of Holstein and Simmental genes: Holstein = 100% Holstein, R1-Holstein —between >50 and <100% Holstein, F1-Crossbreds (50% Holstein/50% Simmental), R1-Simmental—>50 and <100% Simmental, and Simmental = 100% Simmental. **ECM: Energy corrected milk yield, the number of observations is smaller than that for milk yield, because the AMS did not always provide the values for the milk solids.

## Data Availability

The data presented in this study will be made available on reasonable request from the corresponding author.

## Appendix A

**Table A1:** Variance analysis results for the variable body condition score (BCS).

Effect	Num DF *	Den DF **	F Value	Pr > F
Breed	4	151	19.18	<0.0001
Week	16	2450	118.57	<0.0001
Breed × week	64	2450	2.21	<0.0001
Parity	2	551	52.24	<0.0001
Calving year	1	530	64.52	<0.0001
Calving season	5	218	8.73	<0.0001
Data collection season	5	2478	4.55	0.0004
Bedding system	1	252	1.88	0.1712

* Numerator degrees of freedom ** Denominator degrees of freedom.

**Table A2:** Variance analysis results for the variable back fat thickness (BFT).

Effect	Num DF *	Den DF **	F Value	Pr > F
Breed	4	172	1.96	0.1024
Week	16	2466	91.46	<0.0001
Breed × week	64	2467	1.56	0.0031
Parity	2	634	48.58	<0.0001
Calving year	1	600	37.30	<0.0001
Calving season	5	2491	23.49	<0.0001
Data collection season	5	247	3.81	0.0024
Bedding system	1	296	1.81	0.1795

* Numerator degrees of freedom ** Denominator degrees of freedom.

**Table A3:** Variance analysis results for the variable beta-hydroxybutyrate (BHBA).

Effect	Num DF *	Den DF **	F Value	Pr > F
Breed	4	149	2.93	0.0229
Week	16	1801	5.45	<0.0001
Breed × week	64	1799	1.01	0.4630
Parity	2	206	4.37	0.0138
Calving year	1	1144	5.49	0.0193
Calving season	5	230	1.32	0.2573
Data collection season	5	1874	14.84	<0.0001
Bedding system	1	154	0.05	0.8195

* Numerator degrees of freedom ** Denominator degrees of freedom.

**Table A4:** Variance analysis results for the variable non-esterified fatty acids (NEFA).

Effect	Num DF *	Den DF **	F Value	Pr > F
Breed	4	136	3.83	0.0056
Week	16	1811	25.75	<0.0001
Breed × week	64	1807	1.57	0.0029
Parity	2	165	7.85	0.0006
Calving year	1	1058	0.40	0.5271
Calving season	5	238	2.14	0.0618
Data collection season	5	1878	3.75	0.0022
Bedding system	1	139	2.19	0.1411

* Numerator degrees of freedom ** Denominator degrees of freedom.

**Table A5:** Variance analysis results for the variable glucose.

Effect	Num DF *	Den DF **	F Value	Pr > F
Breed	4	144	1.04	0.3904
Week	16	1930	15.90	<0.0001
Breed × week	64	1930	1.07	0.3297
Parity	2	181	23.33	<0.0001
Calving year	1	1139	0.15	0.6965
Calving season	5	2015	3.57	0.0032
Data collection season	5	247	1.23	0.2964
Bedding system	1	147	0.23	0.6358

* Numerator degrees of freedom ** Denominator degrees of freedom.

**Table A6:** Variance analysis results for the variable body weight.

Effect	Num DF *	Den DF **	F Value	Pr > F
Breed	4	152	2.45	0.0482
Week	13	15E3	48.31	<0.0001
Breed × week	52	15E3	8.84	<0.0001
Parity	2	9619	67.96	<0.0001
Calving year	1	8227	109.71	<0.0001
Calving season	5	15E3	86.01	<0.0001
Data collection season	5	251	1.30	0.2649
Bedding system	1	8996	461.46	<0.0001

* Numerator degrees of freedom ** Denominator degrees of freedom.

**Table A7:** Variance analysis results for the variable locomotion score (LS).

Effect	Num DF *	Den DF **	F Value	Pr > F
Breed	4	149	1.23	0.3027
Week	16	2441	4.64	<0.0001
Breed × week	64	2441	1.21	0.1280
Parity	2	399	10.12	<0.0001
Calving year	1	546	5.94	0.0151
Calving season	5	220	2.87	0.0156
Data collection season	5	2491	7.42	<0.0001
Bedding system	1	184	0.04	0.8343

* Numerator degrees of freedom ** Denominator degrees of freedom.

**Table A8:** Variance analysis results for the variable milk yield.

Effect	Num DF *	Den DF **	F Value	Pr > F
Breed	4	138	0.99	0.4154
Week	13	15E3	83.94	<0.0001
Breed × week	52	15E3	2.72	<0.0001
Parity	2	920	129.56	<0.0001
Calving year	1	754	0.48	0.4881
Calving season	5	15E3	17.29	<0.0001
Data collection season	5	226	20.43	<0.0001
Bedding system	1	581	0.76	0.3826

* Numerator degrees of freedom ** Denominator degrees of freedom.

**Table A9:** Variance analysis results for the variable energy corrected milk yield (ECM).

Effect	Num DF *	Den DF **	F Value	Pr > F
Breed	4	142	1.63	0.1689
Week	13	15E3	13.70	<0.0001
Breed × week	52	15E3	2.26	<0.0001
Parity	2	877	131.43	<0.0001
Calving year	1	732	3.50	0.0618
Calving season	5	15E3	8.67	<0.0001
Data collection season	5	230	23.41	<0.0001
Bedding system	1	554	0.14	0.7071

* Numerator degrees of freedom ** Denominator degrees of freedom.

**Table A10:** Variance analysis results for the variable fat (%).

Effect	Num DF *	Den DF **	F Value	Pr > F
Breed	4	147	1.80	0.1328
Week	13	15E3	177.86	<0.0001
Breed × week	52	15E3	1.76	0.0006
Parity	2	739	2.73	0.0660
Calving year	1	653	3.60	0.0582
Calving season	5	15E3	36.07	<0.0001
Data collection season	5	235	4.45	0.0007
Bedding system	1	445	2.43	0.1197

* Numerator degrees of freedom ** Denominator degrees of freedom.

**Table A11:** Variance analysis results for the variable fat (kg/day).

Effect	Num DF *	Den DF **	F Value	Pr > F
Breed	4	147	2.46	0.0479
Week	13	15E3	1.15	0.3085
Breed × week	52	15E3	2.11	<0.0001
Parity	2	886	99.24	<0.0001
Calving year	1	744	4.85	0.0279
Calving season	5	15E3	6.17	<0.0001
Data collection season	5	238	25.32	<0.0001
Bedding system	1	557	0.09	0.7701

* Numerator degrees of freedom ** Denominator degrees of freedom.

**Table A12:** Variance analysis results for the variable protein (%).

Effect	Num DF *	Den DF **	F Value	Pr > F
Breed	4	162	3.55	0.0084
Week	13	15E3	188.03	<0.0001
Breed × week	52	15E3	7.84	<0.0001
Parity	2	1145	6.70	0.0013
Calving year	1	931	4.94	0.0265
Calving season	5	15E3	130.96	<0.0001
Data collection season	5	263	3.63	0.0034
Bedding system	1	749	1.86	0.1734

* Numerator degrees of freedom ** Denominator degrees of freedom.

**Table A13:** Variance analysis results for the variable protein (kg/day).

Effect	Num DF *	Den DF **	F Value	Pr > F
Breed	4	136	0.88	0.4783
Week	13	15E3	23.08	<0.0001
Breed × week	52	15E3	3.08	<0.0001
Parity	2	755	152.45	<0.0001
Calving year	1	648	3.71	0.0546
Calving season	5	15E3	16.72	<0.0001
Data collection season	5	219	14.53	<0.0001
Bedding system	1	464	0.84	0.3592

* Numerator degrees of freedom ** Denominator degrees of freedom.

**Table A14:** Variance analysis results for the variable somatic cell score (SCS).

Effect	Num DF *	Den DF **	F Value	Pr > F
Breed	4	105	1.98	0.1032
Week	13	15E3	20.62	<0.0001
Breed × week	52	15E3	3.34	<0.0001
Parity	2	540	6.69	0.0013
Calving year	1	470	64.65	<0.0001
Calving season	5	15E3	58.77	<0.0001
Data collection season	5	169	80.93	<0.0001
Bedding system	1	325	0.46	0.4980

* Numerator degrees of freedom ** Denominator degrees of freedom.
